# Neutralizing antibody responses elicited by SARS-CoV-2 mRNA vaccination wane over time and are boosted by breakthrough infection

**DOI:** 10.1126/scitranslmed.abn8057

**Published:** 2022-02-15

**Authors:** John P. Evans, Cong Zeng, Claire Carlin, Gerard Lozanski, Linda J. Saif, Eugene M. Oltz, Richard J. Gumina, Shan-Lu Liu

**Affiliations:** ^1^ Center for Retrovirus Research, The Ohio State University, Columbus, OH 43210, USA; ^2^ Department of Veterinary Biosciences, The Ohio State University, Columbus, OH 43210, USA; ^3^ Molecular, Cellular, and Developmental Biology Program, The Ohio State University, Columbus, OH 43210, USA; ^4^ Department of Internal Medicine, Division of Cardiovascular Medicine, The Ohio State University, Columbus, OH 43210, USA; ^5^ Department of Pathology, The Ohio State University, Columbus, OH 43210, USA; ^6^ Center for Food Animal Health, Animal Sciences Department, OARDC, College of Food, Agricultural and Environmental Sciences, The Ohio State University, Wooster, OH 44691, USA; ^7^ Veterinary Preventive Medicine Department, College of Veterinary Medicine, The Ohio State University, Wooster, OH 44691, USA; ^8^ Viruses and Emerging Pathogens Program, Infectious Diseases Institute, The Ohio State University, Columbus, OH 43210, USA; ^9^ Department of Microbial Infection and Immunity, The Ohio State University, Columbus, OH 43210, USA

## Abstract

The waning efficacy of SARS-CoV-2 vaccines, combined with the continued emergence of variants resistant to vaccine-induced immunity, has reignited debate over the need for booster vaccine doses. To address this, we examined the neutralizing antibody response against the spike protein of five major SARS-CoV-2 variants, D614G, Alpha (B.1.1.7), Beta (B.1.351), Delta (B.1.617.2), and Omicron (B.1.1.529), in health care workers (HCWs) vaccinated with SARS-CoV-2 mRNA vaccines. Serum samples were collected pre-vaccination, three weeks post-first vaccination, one month post-second vaccination, and six months post-second vaccination. Minimal neutralizing antibody titers were detected against Omicron pseudovirus at all four time points, including for a majority of patients who had SARS-CoV-2 breakthrough infections. Neutralizing antibody titers against all other variant spike protein-bearing pseudoviruses declined dramatically from one to six months after the second mRNA vaccine dose, although SARS-CoV-2 infection boosted vaccine responses. Additionally, mRNA-1273-vaccinated HCWs exhibited about two-fold higher neutralizing antibody titers than BNT162b2-vaccinated HCWs. Together these results demonstrate possible waning of antibody-mediated protection against SARS-CoV-2 variants that is dependent on prior infection status and the mRNA vaccine received. They also show that the Omicron variant spike protein can almost completely escape from neutralizing antibodies elicited in recipients of only two mRNA vaccine doses.

## Teaser

SARS-CoV-2-specific neutralizing antibodies induced by two doses of mRNA vaccine wane after six months but increase after breakthrough infection.

## INTRODUCTION

Since its emergence in late 2019, the Coronavirus Disease 2019 (COVID-19) pandemic has led to over 323 million confirmed cases and over 5 million deaths as of January 18, 2022 (*1*). In response, several vaccines have been developed against severe acute respiratory syndrome coronavirus 2 (SARS-CoV-2), the causative agent of COVID-19, including two mRNA vaccines, Moderna mRNA-1273 and Pfizer/BioNTech BNT162b2. These highly effective vaccines have helped to stem COVID-19 hospitalizations and deaths. However, the rapid evolution of SARS-CoV-2, combined with waning vaccine efficacy, remains a threat to public health.

Following its introduction into the human population, several SARS-CoV-2 variants of concern (VOCs) have emerged. Soon after initial identification, SARS-CoV-2 acquired a predominant D614G mutation in its spike protein. This mutation leads to enhanced transmissibility, likely due to increased stability of the spike protein, as well as increased viral titers in the nasopharynx and increased infectivity (*2*). As a result, nearly all currently circulating SARS-CoV-2 strains bear the D614G mutation (*3*). However, as greater proportions of the world population acquired immunity against SARS-CoV-2 through infection or vaccination, new VOCs emerged that had reduced susceptibility to antibody-mediated immune responses and continued to become more transmissible (*4*, *5*). One VOC, Alpha (B.1.1.7), is characterized by N-terminal domain (NTD) deletions and a key N501Y mutation in its receptor-binding domain (RBD). The Alpha variant exhibited enhanced transmissibility and rapidly spread from Europe to other parts of the world (*6*). Another VOC to emerge at about the same time was Beta (B.1.351), which is characterized by other NTD mutations and deletions, as well as crucial RBD mutations, including K417N, E484K, and N501Y. Although the Beta variant did not disseminate as widely as the Alpha variant, it harbored strong resistance to vaccine-induced immunity (*7*). The recently emerged Delta (B.1.617.2) variant is characterized by additional NTD alterations together with crucial RBD mutations (L452R and T478K). The Delta variant led to an alarming number of vaccine breakthrough infections worldwide and initially prompted debate about the need for vaccine booster doses. Finally, the Omicron (B.1.1.529) variant, responsible for the ongoing wave of the pandemic, is characterized by an alarming number of mutations, including 16 in the RBD alone. The Omicron variant has produced an unprecedented peak in COVID-19 cases worldwide and appears to continue to exhibit enhanced escape from vaccine-induced immunity (*8*–*10*).

The extent to which the rise in breakthrough infections is caused by increased resistance to vaccine-induced immunity in these variants or to waning durability of immunity conferred by vaccines remains unclear. For the Delta variant, reports from India, where the population was still pursuing mass vaccination efforts, show minor differences in breakthrough infection rates between Alpha and Delta variants. Specifically, BNT162b2 efficacy against symptomatic infection was reported to drop from 93.7% against Alpha to 88.0% against Delta (*11*). However, reports from the U.S. indicate that vaccine efficacy of BNT162b2 against infection with the Delta variant declined from 93% one month after vaccination to 53% at four months (*12*), consistent with an overall waning of vaccine efficacy over time (*13*). A critical goal of this study is to understand how the durability of vaccine efficacy might contribute to rates of breakthrough infections, especially in the context of evolving SARS-CoV-2 variants. Such insights will aid in decision-making for allocating booster doses, including updating recommendations for immunocompromised patients. These data may also help guide the reformulation of future SARS-CoV-2 booster doses. In this study, we examined neutralizing antibody (nAb) titers in 48 vaccinated health care workers (HCWs) against the major SARS-CoV-2 VOCs, as prior studies have shown that nAb titers are a major correlate for protection from SARS-CoV-2 infection (*14*). Serum samples were collected pre-vaccination, one month after the first dose of BNT162b2 or mRNA-1273, and one and six months after the second dose of vaccine.

## RESULTS

### SARS-CoV-2-specific immunity wanes over time after two mRNA vaccine doses.

**Fig. 1.**
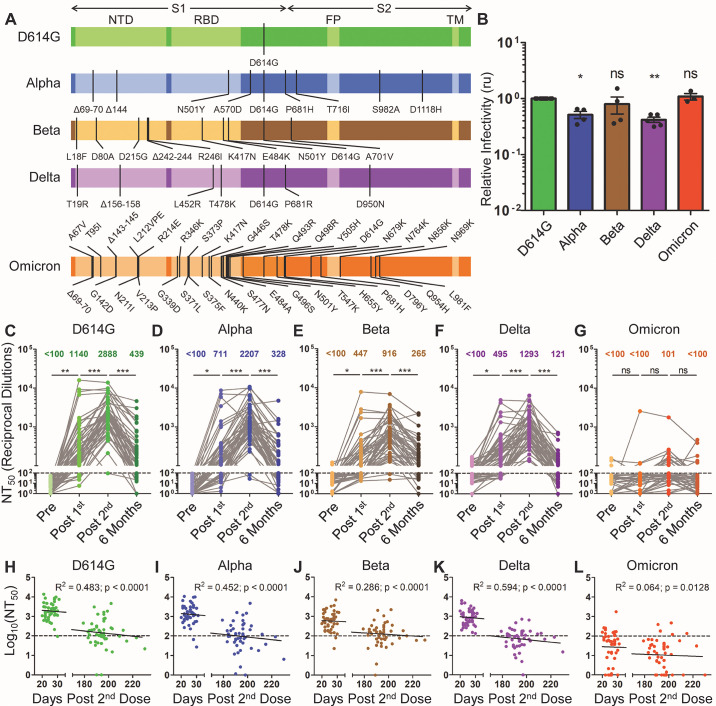
The durability of vaccine-induced immunity wanes over time. *Gaussia* luciferase reporter gene-containing pseudotyped lentiviruses were produced bearing the spike protein from SARS-CoV-2 variants. (**A**) Schematic representations of the SARS-CoV-2 variant spike proteins tested are shown, including D614G, Alpha (B.1.1.7), Beta (B.1.351), Delta (B.1.617.2), and Omicron (B.1.1.529). The schematics highlight the location of the S1 and S2 subunits of the spike protein as well as the N-terminal domain (NTD), receptor-binding domain (RBD), fusion peptide (FP), transmembrane region (TM), and the indicated mutations. (**B**) Pseudotyped lentiviruses were used to infect HEK293T-ACE2 cells. Media was harvested from infected cells 48 hours after infection and assayed for *Gaussia* luciferase activity to determine the relative infectivity of each variant pseudotyped virus; infectivity was measured as luminescence signal relative to D614G signal, relative units (ru). Significance relative to D614G was determined by one-way ANOVA with Bonferroni’s correction (n ≥ 3 biological replicates); error bars represent means ± standard error. (**C to G**) Lentivirus pseudotyped with SARS-CoV-2 spike protein from D614G (C), Alpha (D), Beta (E), Delta (F), and Omicron (G) were neutralized with serum samples from health care workers (HCWs) (n = 48 biological replicates) collected pre-vaccination (Pre), post-vaccination with a first mRNA vaccine dose (Post 1^st^), post-vaccination with a second mRNA vaccine dose (Post 2^nd^), and six months post second dose (Six Months). Neutralization titers 50% (NT_50_) values were determined by least-squares fit non-linear regression. Mean NT_50_ values are shown at the top of the plots, and NT_50_ values below 100 were considered background, as indicated by a dashed line; significance was determined by one-way repeated measures ANOVA with Bonferroni’s correction. (**H to L**) Log_10_-transformed NT_50_ values against D614G (H), Alpha (I), Beta (J), Delta (K), and Omicron (L) variants were plotted against days post-second vaccine dose of sample collection (n = 96 biological replicates). The goodness of fit (R^2^) and p-values are displayed on each plot as determined by least-squares fit linear regression. The dotted lines correspond to the background (NT_50_ < 100). In all cases, *p < 0.05; **p < 0.01; ***p < 0.001; ns: not significant.

We produced pseudotyped lentiviruses expressing a *Gaussia* luciferase reporter gene and bearing SARS-CoV-2 spike proteins derived from D614G, Alpha, Beta, Delta, or Omicron (**Fig. 1A**). D614G was selected to serve as the control virus because this mutation emerged very early in the pandemic, is present in nearly all circulating SARS-CoV-2 variants, and substantially impacts neutralization sensitivity. In light of this, comparisons of neutralization sensitivity to true wild-type SARS-CoV-2 would be confounded by the presence of the D614G mutation in all major SARS-CoV-2 variants. Pseudotyped virus infectivity was then determined by infection of HEK293T cells expressing angiotensin-converting enzyme 2 (HEK293T-ACE2 cells). *Gaussia* luciferase secreted into the media of infected cells was assayed to determine the infectivity of these pseudotyped lentiviruses. We found that pseudotyped virus infectivity was reasonably comparable for all variants, with modest reductions seen for Alpha and Delta compared to D614G (**Fig. 1B**), despite reports of drastically increased transmission and spread for some VOCs, especially the Delta variant (*15*). However, the high transmission rate for the Delta and Omicron variants appears related to faster replication kinetics and improved replication in the nasopharynx for these viruses (*16*, *17*), which would not be reflected in a pseudotyped virus system.

We used our previously reported (*18*, *19*) SARS-CoV-2 spike-pseudotyped lentivirus-based virus neutralization assay to assess nAb titers in HCW samples collected under approved Institutional Review Board (IRB) protocols at The Ohio State University (2020H0228 and 2020H0527). The 48 HCW samples included 22 individuals vaccinated with mRNA-1273 and 26 individuals vaccinated with BNT162b2. The median age of participants was 37 years (interquartile range [IQR] = 31.75-43.25). Samples were collected from HCWs with median time points of 222 days (IQR = 215-225.75) pre-first vaccine dose (Pre), 21 days (IQR = 19.25-23) post-first vaccine dose (Post 1^st^), 26 days (IQR = 22.5-28) post-second vaccine dose (Post 2^nd^), and 194 days (IQR = 190-197.75) post-second vaccine dose (Six Months). According to the pre-determined titer of pseudotyped viruses, we adjusted the volumes of each so that equivalent amounts of infectious virus were used in neutralization assays. HCW serum samples were 4-fold serially diluted, then pseudotyped virus was added for one hour at final dilutions of 1:80, 1:320, 1:1280, 1:5120, 1:20480, and no serum control. For a valid comparison, samples from the same individual collected at different time points were tested side by side on the same plate. HEK293T-ACE2 cells were infected with the virus-serum mixture, and *Gaussia* luciferase activity was assayed 48 hours and 72 hours after infection. Neutralizing titer 50% (NT_50_) values were determined by a nonlinear least-squares regression model.

We compared the strength of the nAb titers over time against all five variants tested. Following the first dose of mRNA vaccine, a strong nAb response was induced among HCWs compared to pre-vaccination across all variants except for Omicron; this was observed despite extensive variation in nAb titers of these individuals, including against D614G (mean = 1140, 95% CI = 317-1963, range = 100-15954) (**Fig. 1C**). A fraction of HCWs exhibited NT_50_ values below the detection limit (NT_50_ less than 100) against D614G (7 of 48), Alpha (17 of 48), Beta (22 of 48), and Delta (14 of 48) following the first dose of vaccine (**Fig. 1C to F**). In contrast, a striking 93.8% (45 of 48) fell below the detection limit for the Omicron variant (**Fig. 1G**). The below-detection rates fell to 0.0% (0 of 48) to 4.2% (2 of 48) for all variants except Omicron following a second vaccine dose, with a 2 to 3-fold increase in mean nAb titers compared to the first dose (p < 0.001 for D614G, Alpha, Beta, and Delta) (**Fig. 1C to F**). Once again, 25.0% (12 of 48) of samples remained below the detection limit for Omicron for the post-second dose, and the overall increase in NT_50_ was not significant compared to the first dose (p > 0.05, **Fig. 1G**). Notably, four HCWs with higher nAb titers after the first vaccine dose did not show an increase, but rather a plateau or slight decline in nAb titers following the second dose (**Fig. 1C to F**). These four individuals included one that had antibodies against the SARS-CoV-2 nucleocapsid (N) protein positive pre-vaccination and three that had anti-N protein antibodies after their first vaccine dose, which indicated infection either prior to or shortly after their first vaccine dose. Of note, the single patient exhibiting strong neutralization of Omicron had antibodies against N protein after the first vaccine dose (**Fig. 1G**), suggesting breakthrough infection. Overall, we found that, following two vaccine doses, the Alpha, Beta, Delta, and Omicron VOCs exhibited a 1.3- (p < 0.01), 3.2- (p < 0.001), 2.2- (p < 0.001), and 28.6-fold (p < 0.001) lower NT_50_ values compared to D614G, respectively (**Fig. 1C to G**). Critically, six months post-vaccination, there was a 3.5 to 10.7-fold reduction in nAb titers against the Alpha, Beta, and Delta variants, with 37.5% (18 of 48) to 56.3% (27 of 48) of HCWs exhibiting NT_50_ values below the limit of detection (**Fig. 1C to F**). Consistent with earlier time points, minimal responses against Omicron were detected in samples collected six months post-second vaccination, with 89.6% (43 of 48) of HCWs falling below the limit of detection (**Fig. 1G**). The mean NT_50_ values for Alpha, Beta, Delta, and Omicron variants at six months were 1.3-, 1.7-, 3.6-, and 10.2-fold lower than that of D614G, respectively (**Fig. 1C to G**).

We also examined the correlation between time post-second dose and log_10_ transformed NT_50_ values. We found a statistically significant association (p < 0.001) between these values for all variants except Omicron (**Fig. 1H to L**). This corresponded to an approximately 10-fold decline in NT_50_ for D614G, Alpha, and Delta (R^2^ = 0.0452 to 0.594, p < 0.0001) every 22 weeks compared with Beta (R^2^ = 0.286, p < 0.001) every 37 weeks (**Fig. 1H to K**). For Omicron, only a weak, but significant, correlation was observed (R^2^ = 0.064, p = 0.0128) (**Fig. 1L**), likely due to the overall weak responsiveness against Omicron at all four time points.

### Breakthrough SARS-CoV-2 infection boosts nAb responses.

**Fig. 2.**
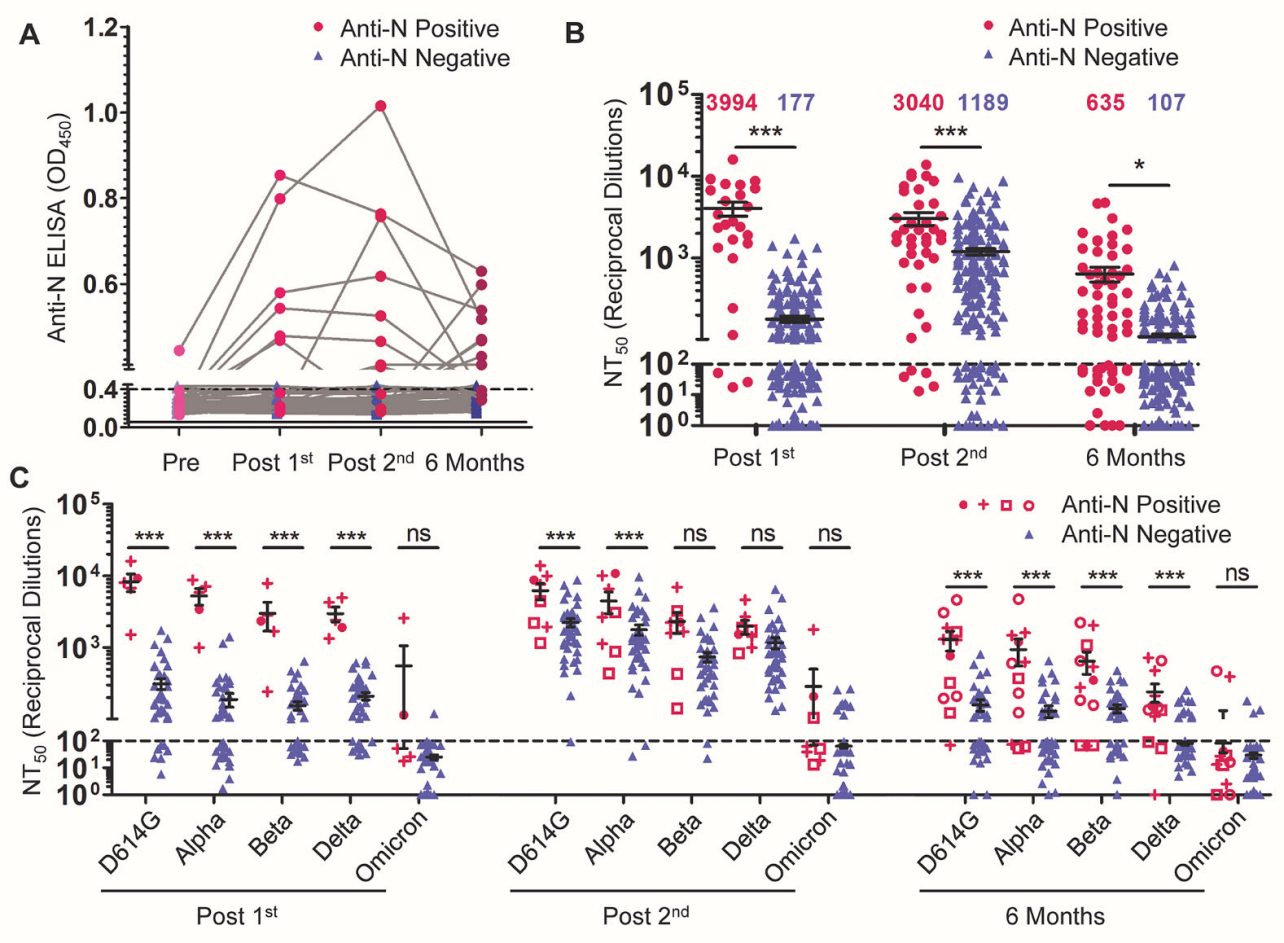
The durability of the nAb response is influenced by prior COVID-19 infection and mRNA vaccine type. COVID-19 status was determined by anti-N protein ELISA. Individuals were characterized as anti-N protein positive (optical density [OD]_450_ > 0.4 at any time point; n = 12) or anti-N protein negative (OD_450_ < 0.4 for all time points; n = 36). (**A**) ELISA optical density at 450nm (OD_450_) are shown for anti-N protein positive and anti-N protein negative HCWs. The dashed line indicates the cut-off of 0.4. (**B**) Comparisons of NT_50_ values between anti-N protein positive and anti-N protein negative HCWs are shown for the indicated time points. NT_50_ values against all variants were combined and plotted at post-first vaccine dose (n = 25 anti-N protein positive; n = 215 anti-N protein negative), post-second vaccine dose (n = 40 anti-N protein positive; n = 200 anti-N protein negative), and six months post-second vaccine dose (n = 60 anti-N protein positive; n = 180 anti-N protein negative). Significant differences were determined by two-way ANOVA with Bonferroni’s correction. (**C**) Comparisons of NT_50_ values against different variants between anti-N protein positive and anti-N protein negative HCWs are shown for the indicated time points. For anti-N protein positive HCWs, timing of first anti-N protein positive sample is distinguished as: pre-vaccination (solid magenta circle ●, n = 1); post-first dose (magenta cross +, n = 4); post-second dose (magenta open square □, n = 3); and six months-post second (magenta open circle ○, n =4). These are plotted alongside anti-N protein negative HCWs (all shown in green blue ▲) for three time points: post-first vaccine dose (n = 5 anti-N protein positive; n = 43 anti-N protein negative), post-second vaccine dose (n = 8 anti-N protein positive; n = 40 anti-N protein negative), and six months post-second vaccine dose (n = 12 anti-N protein positive; n = 36 anti-N protein negative). Significant differences were determined by two-way repeated-measures ANOVA with Bonferroni’s correction. For panels B and C, error bars indicate means ± standard errors, and the dashed horizontal line indicates the limit of detection (NT_50_ < 100). In all cases, *p < 0.05; **p < 0.01; ***p < 0.001; ns: not significant.

Prior COVID-19 status is a critical parameter for the nAb response to vaccination (*20*). Of the 48 HCWs examined, one HCW was positive for antibodies against the SARS-CoV-2 N protein (anti-N protein positive) by enzyme-linked immunosorbent assay (ELISA) before vaccination—indicating a SARS-CoV-2 infection prior to vaccination. Four HCWs were anti-N protein positive at their post-first vaccine dose sample, indicating a SARS-CoV-2 infection before or within three weeks following their first vaccine dose. Three HCWs were anti-N protein positive at their post-second vaccine dose sample, indicating a SARS-CoV-2 infection more than three weeks after their first dose but within four weeks of their second dose. Finally, samples from four HCWs collected six months after their second vaccine were anti-N protein positive, indicating a SARS-CoV-2 infection more than four weeks after their second dose. In total, 12 of 48 individuals were infected by SARS-CoV-2 at different phases of vaccination (**Fig. 2A**). At the time of pre-vaccination sample collection, D614G was the major circulating SARS-CoV-2 variant, whereas at the time of post-first dose and post-second dose sample collections, D614G and Alpha were the major circulating variants. The Delta variant was the dominant circulating strain at the six-month time point. Notably, not all patients remained anti-N protein positive, but were still considered to have been infected for the purpose of analysis.

Following the first vaccine dose, anti-N protein positive HCWs (n = 5) exhibited a 22.6-fold higher mean NT_50_ value (p < 0.001) against all five viruses compared to the anti-N protein negative HCWs (n = 43) (**Fig. 2B**). This difference fell to 2.6-fold following a second vaccine dose (p < 0.001) (n = 8 anti-N protein positive HCWs; n = 40 uninfected anti-N protein negative HCWs) (**Fig. 2B**). However, at six months post-vaccination, anti-N protein positive HCWs (n = 12) exhibited 5.9-fold higher NT_50_ values than anti-N protein negative HCWs (n = 36) for all variants (p = 0.033) (**Fig. 2B**). Interestingly, we found that the differences in NT_50_ between anti-N protein positive and negative HCWs were greater for D614G and Alpha compared with the Beta, Delta, and Omicron variants, possibly due to the stronger neutralization resistance of the latter VOCs (**Fig. 2C**). Notably, for anti-N protein negative HCWs, between 41.7% (15 of 36) and 66.7% (24 of 36) of individuals exhibited nAb titers against all variants except Omicron that were below the detection limit at six months, in contrast to anti-N protein positive individuals, who were between 8.3% (1 of 12) and 25.0% (3 of 12) (**Fig. 2C**). The nAb titers for Omicron were too low at all time points to compare the anti-N positive and anti-N protein negative HCWs (**Fig. 2C**).

### Vaccine type and sex, but not age, impact nAb responses elicited by SARS-CoV-2 vaccination.

**Fig. 3.**
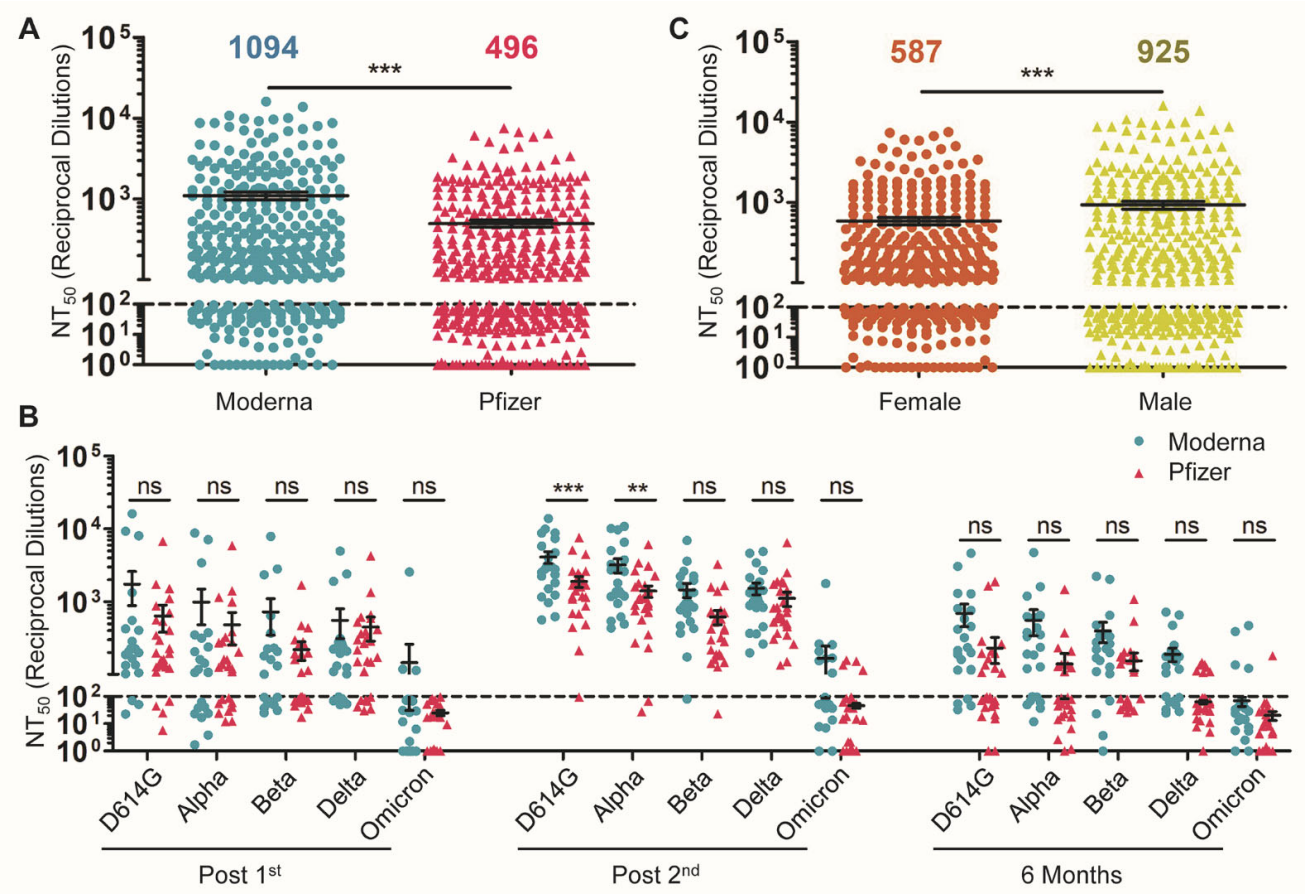
nAb response is influenced by vaccine type and sex. (**A**) HCWs were divided by types of mRNA vaccine received, either Moderna mRNA-1273 (n = 22) or Pfizer/BioNTech BNT162b2 (n = 26), and NT_50_ values were combined for all variants at all time points. Significance between vaccine type was determined by unpaired two-tailed *t* test with Welch’s correction and means are indicated at the top of the plot. (**B**) NT_50_ values of serum samples from individuals vaccinated with Moderna mRNA-1273 (n = 22) or Pfizer/BioNTech BNT162b2 (n = 26) were grouped by variant and time point, with significance determined by two-way repeated-measures ANOVA with Bonferroni’s correction. (**C**) NT_50_ values against all variants at post-first dose, post-second dose, and six months post-second dose combined were compared for male (n = 390) and female (n = 330) participants; mean NT_50_ values are displayed at the top of the plot, with significance determined by unpaired, two-tailed *t* test with Welch’s correction. For all panels, error bars indicate means ± standard errors, and the dashed horizontal line indicates the limit of detection (NT_50_ < 100). In all cases, *p < 0.05; **p < 0.01; ***p < 0.001; ns: not significant.

We further examined the difference in nAb durability between HCWs who received either the Moderna mRNA-1273 vaccine or the Pfizer/BioNTech BNT162b2 vaccine. Across all variants over the full-time course, we observed that mRNA-1273 elicited an overall 2.2-fold higher nAb response than BNT162b2 (p < 0.001) (**Fig. 3A**). In particular, following two vaccine doses, mRNA-1273 vaccinated HCWs exhibited significantly higher nAb titers than BNT162b2-vaccinated HCWs for the D614G (p < 0.001) and Alpha (p < 0.01) variants, respectively (**Fig. 3B**). However, by six months post-second vaccine dose, no significant differences were observed between mRNA-1273 and BNT162b2 vaccinated HCWs (p > 0.05; **Fig. 3B**). Again, because most of the HCW samples exhibited an NT_50_ value below the limit of detection against Omicron, a comparison between mRNA-1273 and BNT162b2 was not informative (**Fig. 3B**).

We examined additional factors that may also contribute to the strength and duration of the nAb response to vaccination, including age and sex. We found that male HCWs exhibited significantly higher NT_50_ titers (p < 0.001) compared to females against all five variants over the post-vaccination time points (**Fig. 3C**). However, this difference was not significant when examined for individual viruses at individual time points (p > 0.05, **fig. S1**). Indeed, studies on sex-based differences in response to COVID-19 mRNA-vaccination have yielded conflicting results (*21*, *22*), although females seem to induce more robust humoral immune responses following vaccination (*23*). We observed no significant correlation for age and NT_50_ against D614G at any time point (p > 0.05, **fig. S2**), which could be due to the relatively young pool of individuals in this cohort (median age 37, maximum age of 61) compared to older adults in a prior mRNA vaccine efficacy study (*24*).

## DISCUSSION

Here we report a decline of SARS-CoV-2 nAb at six months post-mRNA vaccination and present several key factors that may contribute to these kinetics. Most importantly, we observed a drop in nAb titers from one month to six months after the second vaccine dose, with 56.3% and 89.6% of HCWs exhibiting NT_50_ values below the limit of detection against Delta and Omicron at six months, respectively. Interestingly, we found that the NT_50_ values of anti-N protein positive HCWs were approximately six times higher than that of anti-N protein negative HCWs at six months post-vaccination, with about 30% below the background of detection for the former compared to about 60% for the latter. This suggests that breakthrough infection can improve the durability of the SARS-CoV-2 nAb response, which is consistent with data collected after administration of mRNA vaccine booster doses (*25*). We observed a profound escape of the Omicron variant from mRNA vaccine-induced immunity, even at 3 to 4 weeks after the second dose of mRNA vaccination. Further, this escape was not rescued in most HCWs by breakthrough infection. Together, these results, along with recent reports of enhanced neutralization of Omicron following booster dose administration (*9*, *26*), support the need for boosters and alternative vaccination strategies to achieve long-term protection from infection with SARS-CoV-2.

Additionally, we observed that individuals vaccinated with BNT162b2 exhibited lower nAb titers than individuals vaccinated with mRNA-1273. However, the trend for declining nAb titers was consistent for both vaccines. Thus, both mRNA vaccines require booster doses to maintain protective nAb titers, although the waning of nAb responses likely occurs over a relatively long period for mRNA-1273. Further examination of the durability of cellular immunity following mRNA vaccination is warranted, as this more persistent immune response may limit the rates of hospitalization and death, which remain low for mRNA-vaccinated individuals (*27*).

In this study, we found that all four VOCs consistently had reduced NT_50_ values compared to D614G at all time points, with the Omicron variant showing the most pronounced nAb resistance, followed by the Beta and Delta variants. These results are consistent with preliminary reports from our group and others (*9*, *19*, *28*–*30*). However, we found that the Delta variant exhibited comparable or even higher resistance to nAbs than the Beta variant for samples collected at six months post-vaccination. The more modest drop in NT_50_ values at six months for the Beta variant was unclear but could be the result of this variant’s pre-existing resistance to neutralization following the second dose of vaccination. Further, the more dramatic decline in nAb titers against the Delta variant could be attributed to a lower frequency and durability of nAb-producing plasma cells. As reported by others, the rampant spread of the Delta variant in vaccinated and unvaccinated populations is likely related to other factors, such as its high replication kinetics and transmissibility (*31*) coupled with its comparable neutralization resistance.

The major limitation of this study is its reliance on nAb data. Although nAb titers remain a crucial predictor of protection from viral infection, no clear cut-off is available to define an NT_50_ threshold that indicates protective immunity. Further, other immune factors, including T cell immunity, may have a greater impact on protection from severe disease. Additionally, this study utilizes a pseudotyped virus system rather than authentic SARS-CoV-2. Although our pseudotyped virus system has been shown to correlate well with authentic virus systems for determine neutralization titers (*18*), it is not a perfect recapitulation of the authentic virus system.

Overall our data highlight the need for booster vaccinations as the nAb response elicited by a two-dose vaccine regimen wanes over time and is largely ineffective against the Delta and Omicron variants by 6 months. Additionally, although breakthrough infection can boost nAb responses, it appears largely ineffective for providing protection from Omicron, at least for individuals infected before the Omicron wave. Finally, further study is warranted on the durability of the nAb response in booster vaccine recipients. As SARS-CoV-2 variants continue to emerge, understanding the interplay between vaccine durability and virus evolution will be critical for revising vaccination strategies and formulations as we seek to curb the ongoing pandemic.

## MATERIALS AND METHODS

### Study Design

De-identified vaccinated HCW’s serum samples were collected under approved IRB protocols (2020H0228 and 2020H0527). These 48 HCWs ranged in age from 22 to 61 years (median = 37; IQR = 31.75 to 43.25) and included 26 male and 22 female HCWs. HCWs were vaccinated with either the Moderna mRNA-1273 vaccine (n = 22) or the Pfizer/BioNTech BNT162b2 vaccine (n = 26). Serum samples were collected from HCWs at four time points, with a median time point of 222 days (IQR = 215 to 225.75) pre-first vaccine dose (Pre), 21 days (IQR = 19.25 to 23) post-first vaccine dose (Post 1^st^), 26 days (IQR = 22.5 to 28) post-second vaccine dose (Post 2^nd^), and 194 days (IQR = 190 to 197.75) post-second vaccine dose (6 Months). HCWs received their second vaccine dose between January and February of 2021. No other demographic information was collected on these HCWs. HCW COVID-19 status was determined by anti-N protein ELISA. Of the 48 HCWs examined, one was anti-SARS-CoV-2 N protein positive by ELISA for their pre-vaccination sample. Additionally, four HCWs became anti-N protein positive for their post-first vaccine dose sample, three became positive for their post-second vaccine dose sample, and four became positive for their six-month vaccine sample. This indicated that these 12 individuals were infected by SARS-CoV-2 at different phases after vaccination. HCW serum samples were then assessed for nAb titers against SARS-CoV-2 variant spike protein-pseudotyped lentiviruses. Individual-level NT_50_ values are presented in data file S1.

### Constructs for Pseudotyped Lentivirus Production

The pseudotyped lentiviruses were produced using a previously reported protocol using the pNL4-3-HIV-1-inGluc vector (*18*, *19*, *32*–*34*). This vector is a pNL4-3-HIV-1 ΔEnv construct and contains a *Gaussia* luciferase reporter gene with a cytomegalovirus (CMV) promoter, both oriented in an antisense orientation relative to the HIV-1 genome. This *Gaussia* luciferase reporter gene contains a sense orientation intron, which prevents the expression of *Gaussia* luciferase in the virus-producing cells. However, after the intron is spliced from full-length virus genomes upon integrated into target cells, target cells can produce *Gaussia* luciferase, which is secreted in mammalian cell culture (*35*). Constructs encoding N- and C-terminal flag-tagged SARS-CoV-2 spike protein for each variant — D614G, Alpha (B.1.1.7), Beta (B.1.351), Delta (B.1.617.2), and Omicron (B.1.1.529; also known as BA1.1 containing the R346K mutation) — were synthesized and cloned into pcDNA3.1 vector using KpnI/BamHI restriction enzyme cloning by GenScript Biotech.

### Cell Lines and Maintenance

HEK293T cells (CRL-11268, CVCL_1926, American Type Culture Collection [ATCC]) and HEK293T-ACE2 cells (NR-52511, the Biodefense and Emerging Infections Research Resources Repository [BEI Resources], ATCC) were maintained in Dulbecco’s Modified Eagles Medium (Gibco, 11965-092, Thermo Fisher Scientific) supplemented with 10% (v/v) fetal bovine serum (F1051, Sigma-Aldrich) and 1% (v/v) penicillin/streptomycin (SV30010, HyClone Laboratories Inc.). Cells were maintained at 37°C and 5% CO_2_.

### Pseudotyped Lentivirus Production and Titering

Pseudotyped lentivirus was produced by co-transfection of HEK293T cells with pNL4-3-HIV-1-inGluc and pcDNA3.1 vector expressing the spike protein of interest (D614G, B.1.1.7, B.1.351, B.1.617.2, or B.1.1.529) in a 2:1 ratio using polyethylenimine (PEI) transfection. Virus was collected at 24 hours, 48 hours, and 72 hours after transfection, then was pooled and stored at -80°C. To determine relative titers of harvested virus, the pseudotyped virus for each of the SARS-CoV-2 variants was used to infect HEK293T-ACE2 cells. Then, 48 hours and 72 hours after infection, *Gaussia* luciferase activity in the media of infected cells was measured. Twenty μL of cell culture media and 20 μL of *Gaussia* luciferase substrate (0.1M Tris [T6066, Millipore Sigma] pH 7.4, 0.3M sodium ascorbate [S1349, Spectrum Chemical Mfg. Corp.], and 10 μM coelenterazine [CZ2.5, Gold Biotechnology]) were combined in white polystyrene 96-well plates. Luminescence was immediately measured by a BioTek Cytation5 plate-reader.

### Virus Neutralization Assays

Virus neutralization assays were performed as previously reported (*18*, *19*, *34*). In a 96-well format, HCW serum was 4-fold serially diluted, and 100 μL of pseudotyped virus was added to each well (final dilutions of 1:80, 1:320, 1:1280, 1:5120, 1:20480, and no serum). To ensure comparable results between SARS-CoV-2 variants, equivalent amounts of infectious virus were used based on the pre-determined virus titers. Virus was incubated with HCW serum for 1 hour at 37°C, followed by infection of HEK293T-ACE2 cells seeded on a 96-well polystyrene tissue culture plate. *Gaussia* luciferase activity in cell culture media was then assayed 48 hours and 72 hours after infection, as described above. Neutralizing titer 50% (NT_50_) for each serum sample was determined by non-linear regression with least-squares fit in GraphPad Prism 5 (GraphPad Software).

### Anti-N protein ELISA:

Anti-N protein ELISA was performed as previously reported (*18*). ELISA was performed by using the Epitope Diagnostics, Inc. (EDI) Novel Coronavirus COVID-19 N protein IgG ELISA Kit (KT-1032, EDI) following the manufacturer’s protocol. Briefly, 100 μL of a 1:100 dilution of HCW serum was added to microplates coated with SARS-CoV-2 N antigen and incubated for 30 min. Plates were then washed and treated with 100 μL of horseradish peroxidase (HRP)-labeled anti-human-IgG antibody (31220, EDI) for 30 min. Then plates were washed, and 100 μL of ELISA HRP substrate (10020, EDI) was added and incubated for 20 min before 100 μL of stop solution (10030, EDI) was added. Absorbance at 450 nm was read by a Biotek Synergy/HTX Multi mode plate reader using Gen 5 software.

### Statistical Analyses:

Statistical analysis was done with GraphPad Prism 5. Comparisons between multiple groups were made using one-way analysis of variance (ANOVA) with Bonferroni’s multiple testing correction (data in Fig. 1B and Fig. 2B) or one-way repeated measures ANOVA with Bonferroni’s multiple testing correction (data in Fig. 1, C to G). For comparisons between two treatments across multiple groups, a two-way ANOVA with Bonferroni’s multiple testing correction was used (data in Fig. 2C, Fig. 3B, and fig. S1). For comparisons between two groups, an unpaired, two-tailed student’s *t* test with Welch’s correction was used (data in Fig. 3, A and C). For correlative analyses between two continuous variables, a linear regression model with least-squares fit was used with log_10_ transformed NT_50_ values to better approximate linearity (data in Fig. 1, H to L, and fig. S2). An alpha value of 0.05 was considered significant throughout.

## Supplementary Materials

### Other Supplementary Material for this manuscript includes the following:

MDAR Reproducibility Checklist
