# Supplementary material for: Neutralizing antibody responses elicited by SARS-CoV-2 mRNA vaccination wane over time and are boosted by breakthrough infection
Source: Sci Transl Med. 2022 Feb 15:eabn8057. doi: 10.1126/scitranslmed.abn8057 (PMC8939766; doi:10.1126/scitranslmed.abn8057)
Supplement: Supplementary file 1 — Figs. S1 and S2 [file scitranslmed.abn8057_sm.pdf]

Science Translational Medicine
AAAS

Supplementary Materials for

Neutralizing antibody responses elicited by SARS-CoV-2 mRNA vaccination wane over time and are boosted by breakthrough infection

John P. Evans *et al.*

Corresponding author: Shan-Lu Liu, Liu.6244@osu.edu

DOI: 10.1126/scitranslmed.abn8057

The PDF file includes:

Figs. S1 and S2

Other Supplementary Material for this manuscript includes the following:

MDAR Reproducibility Checklist
Data file S1

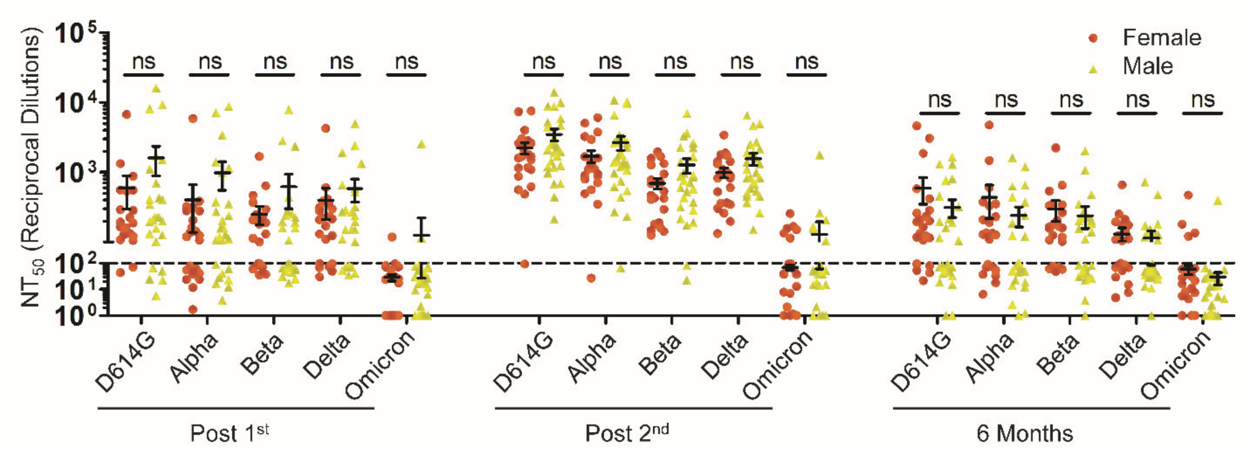

Fig. S1. Sex did not impact neutralizing antibody responses elicited by mRNA vaccination. Neutralizing titer 50% (NT_{50}) values for male (n = 26) and female (n = 22) health care workers (HCWs) were measured against indicated spike protein-pseudotyped lentiviruses at three time points post-vaccination; the horizontal dashed line indicates the limit of detection (NT_{50} < 100). Significance was determined by two-way repeated-measures analysis of variance (ANOVA) with Bonferroni's correction; error bars represent means ± standard errors. ns: not significant.

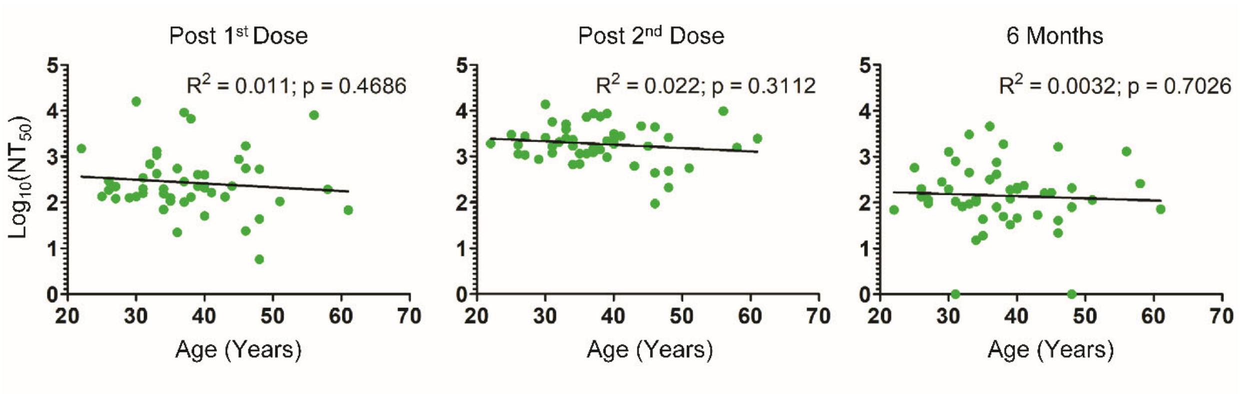

Fig. S2. Age did not impact neutralizing antibody responses elicited by mRNA vaccination. Log_{10}-transformed NT_{50} titers against D614G-SARS-CoV-2 spike protein-pseudotyped lentivirus were plotted against age (in years) for HCW samples (n=48) collected post-first vaccine dose (left), post-second vaccine dose (middle), and six months post-second vaccine dose (right), with goodness of fit (R^2) and significance (p-value) displayed as determined by linear regression with least-squares residual fit.

Data file S1. NT_{50} values. NT_{50} values are included for 48 HCWs for sample collections pre-vaccination, post-first mRNA vaccine dose, post-second mRNA vaccine dose, and 6 months post-second mRNA vaccine dose against pseudotyped lentivirus bearing the S of the D614G, Alpha, Beta, Delta, and Omicron SARS-CoV-2 variants. Additionally, the timing of anti-N positivity for 12 HCWs is indicated. The second tab includes descriptive documentation of column headers.
